# Melamine Induces Oxidative Stress in Mouse Ovary

**DOI:** 10.1371/journal.pone.0142564

**Published:** 2015-11-06

**Authors:** Xiao-Xin Dai, Xing Duan, Xiang-Shun Cui, Nam-Hyung Kim, Bo Xiong, Shao-Chen Sun

**Affiliations:** 1 College of Animal Science and Technology, Nanjing Agricultural University, Nanjing, 210095, China; 2 Department of Animal Sciences, Chungbuk National University, Cheongju, 361–763, Korea; Institute of Zoology, Chinese Academy of Sciences, CHINA

## Abstract

Melamine is a nitrogen heterocyclic triazine compound which is widely used as an industrial chemical. Although melamine is not considered to be acutely toxic with a high LD50 in animals, food contaminated with melamine expose risks to the human health. Melamine has been reported to be responsible for the renal impairment in mammals, its toxicity on the reproductive system, however, has not been adequately assessed. In the present study, we examined the effect of melamine on the follicle development and ovary formation. The data showed that melamine increased reactive oxygen species (ROS) levels, and induced granulosa cell apoptosis as well as follicle atresia. To further analyze the mechanism by which melamine induces oxidative stress, the expression and activities of two key antioxidant enzymes superoxide dismutase (SOD) and glutathi-one peroxidase (GPX) were analyzed, and the concentration of malondialdehyde (MDA) were compared between control and melamine-treated ovaries. The result revealed that melamine changed the expression and activities of SOD and GPX in the melamine-treated mice. Therefore, we demonstrate that melamine causes damage to the ovaries via oxidative stress pathway.

## Introduction

Till September 2008, more than 294,000 children had been found affected by melamine- contaminated milk powder in China [1]. Over 50,000 were children with serious illness and at least six were dead [1–3]. Thus melamine contamination has attracted intense panic and widespread concerns.

Melamine (2, 4, 6-triamino-1, 3, 5-triazine), a chemical material, is most commonly used in plastics, coatings, commercial filters, glues, dishware, and flame retardants [4,5]. Previous studies have demonstrated that melamine alone is not considered acutely toxic with a high LD_50_ in animals, and the oral LD_50_ ranges from 3.2 g/kg to 7.0 g/kg in mice [6], which is believed to have low acute oral toxicity. However, long-term exposure to melamine could lead to infertility and fetal toxicity in animals. Melamine has been reported to induce oxidative stress and cause oxidative disorders in the NRK-52e cells and PC12 cells [7,8].

Oxidative stress behaves imbalancedly between the production of reactive oxygen species (ROS) and numerous enzymatic and non-enzymatic antioxidants [9,10]. Excessive ROS can damage lipids, fatty acids, proteins and oxidative DNA, leading to structural and functional disruption of the cell membrane, inactivation of enzymes and cell death [11–13].

ROS, the oxygen containing molecules, are classified into three types including radical(O2−), hydroxyl radical (OH) and non-radical (H_2_O_2_) [14]. In cells, the antioxidant defense machinery has three major antioxidant enzymes, including glutathione peroxidases (GPX), superoxide dismutase (SOD) and catalase (CAT), as well as numerous non-enzymatic antioxidants, such as reduced and oxidized glutathione [9]. Among three enzymatic antioxidants, SOD catalyzes the conversion of O_2_- to H_2_O_2_, while GPX and CAT further degrade the end product to water (H_2_O) [10,15].

Recent research on the toxicity of melamine has mainly focused on renal toxicity due to the crystal formation and lesions to the liver [16,17]. However, it has been less extensively investigated on the reproductive toxicity in female animals. Compared with other systems, the reproductive system is more sensitive to toxic chemicals [18], and it has been shown that oxidative stress can lead to a number of reproductive diseases [15,19]. Our most recent report indicates that melamine disrupts mouse oocyte maturation via perturbing the cytoskeleton and epigenetic modifications [20]. Here, we further show the effect of melamine on the follicle development via the oxidative stress pathway, providing additional evidence regarding the toxicity of melamine on the female reproductive system in mammals.

## Result

### Effect of Melamine on ROS Levels in Oocytes

**Fig 1:**
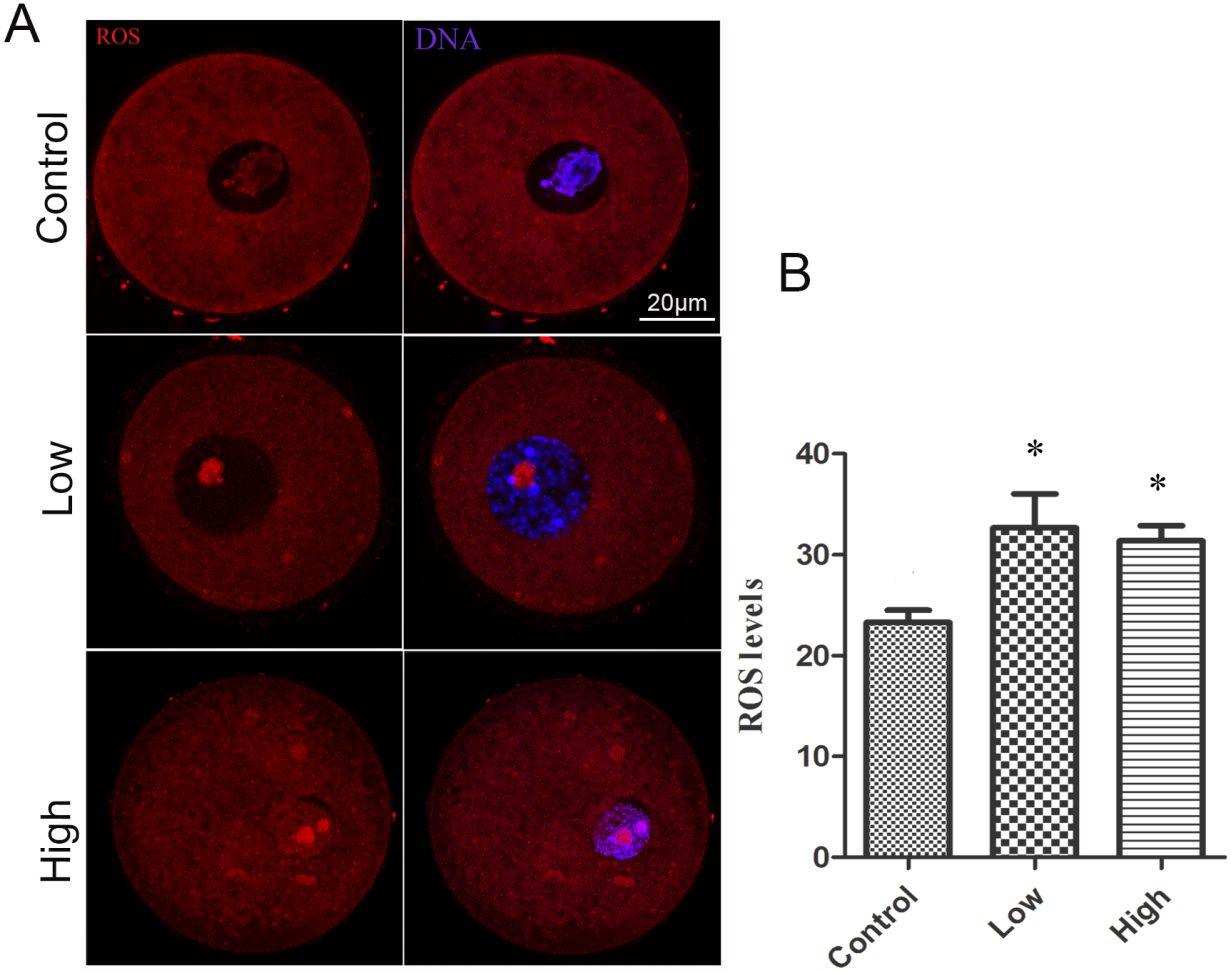
Effect of melamine on ROS levels in oocytes. (A) ROS were stained with Intracellular ROS Red Fluorescence Determination Kit (GENMED, Shanghai, China) (white arrow) and examined by fluorescent microscopy. (B) Average ROS fluorescence intensities in the nucleus were determined in mouse oocytes. The level of ROS was significantly increase in treatment groups (10 mg/kg/day: n = 11, 50 mg/kg/day: n = 12) compared to control group (n = 11). *Significantly different (P <0.05).

Elevated ROS levels are a direct indicator of oxidative stress in biological systems. To examine whether melamine induces oxidative stress in mouse oocytes, we measured the ROS levels of oocytes by immunofluorescence. As shown in Fig 1, compared to the control GV oocytes, the treatment oocytes had big accumulated spots inside the nuclear and exhibited significantly increased levels of ROS in statistics (P < 0.05).

### Melamine Treatment Affects Gene Expression of Antioxidant Enzymes

**Fig 2:**
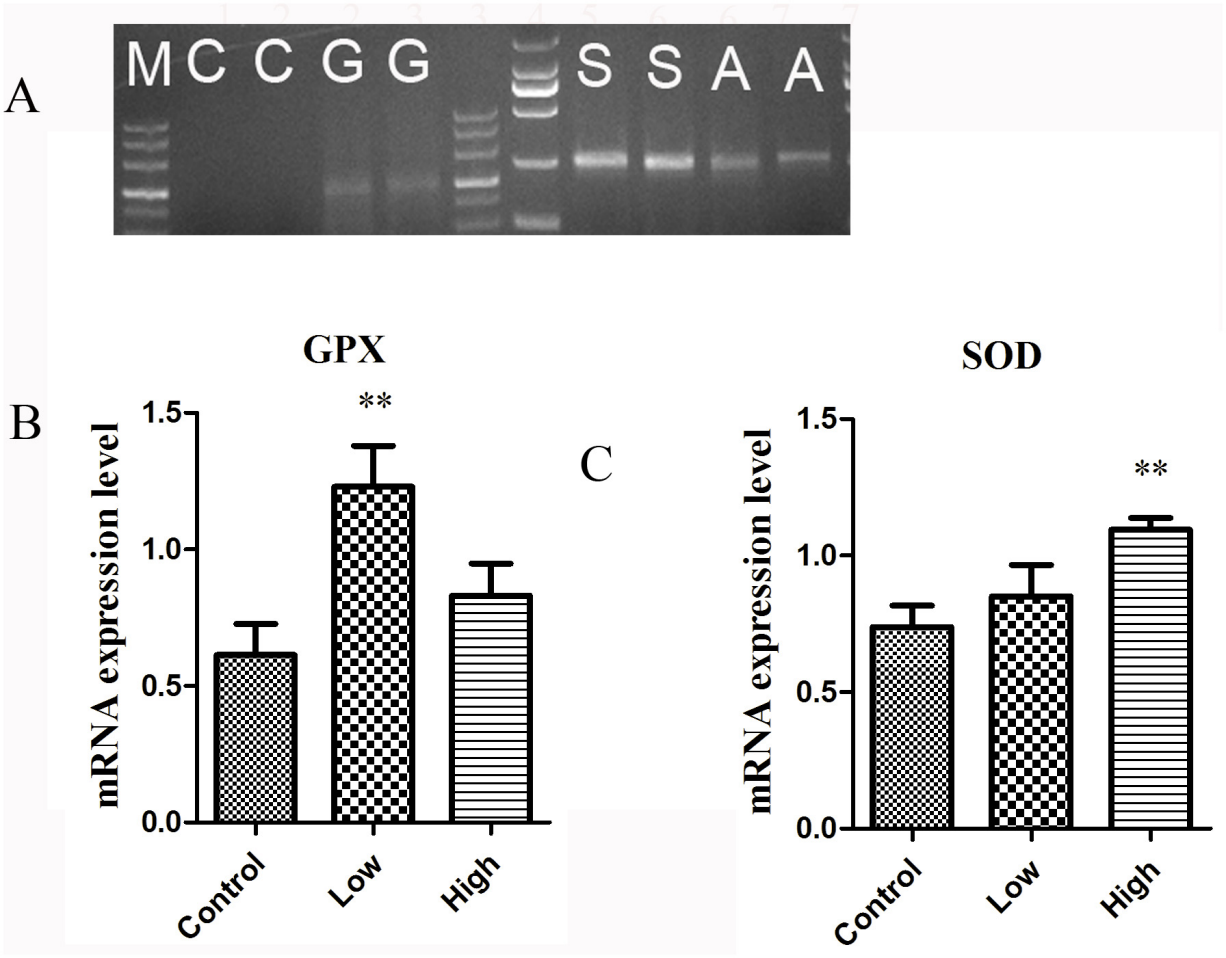
Effect of melamine exposure on the expression enzymes genes. (A) Transcripts encoding for GPX, SOD (lanes G, S respectively) were detected at the GV stage in mouse oocytes. Transcripts encoding for Cat (lane C) were not observed at the GV stage. (B) The expression levels of GPX, SOD in oocytes by Quantitative PCR analysis. In the low group, GPX expression levels were significantly enhanced compare to control group. (C) About SOD expression levels, in high-dose group were remarkably increase than that of control group and there was no significant difference between low-dose group and control group. Data show the means ± SE from at least three separate experiments. ** P < 0.01 compared to control.

Because melamine induced oxidative stress in oocytes, experiments were conducted to determine if it could change the expression of antioxidant enzymes that are required for detoxification and decrease of the ROS levels. To do this, transcripts of GPX, SOD and CAT were detected in mouse GV oocytes. As shown in Fig 2, the expression of GPX in the low-dose treatment group was remarkably higher than that of control group (P < 0.01), but the high-dose treatment group was similar to that of control. As for the expression of SOD, the high-dose group was significantly higher than the control group (P < 0.01), and there was no significant difference between low-dose and control groups. However, CAT transcripts could not be detected in mouse oocytes.

### Melamine Treatment Changes the Activity of Antioxidant Enzymes

**Fig 3:**
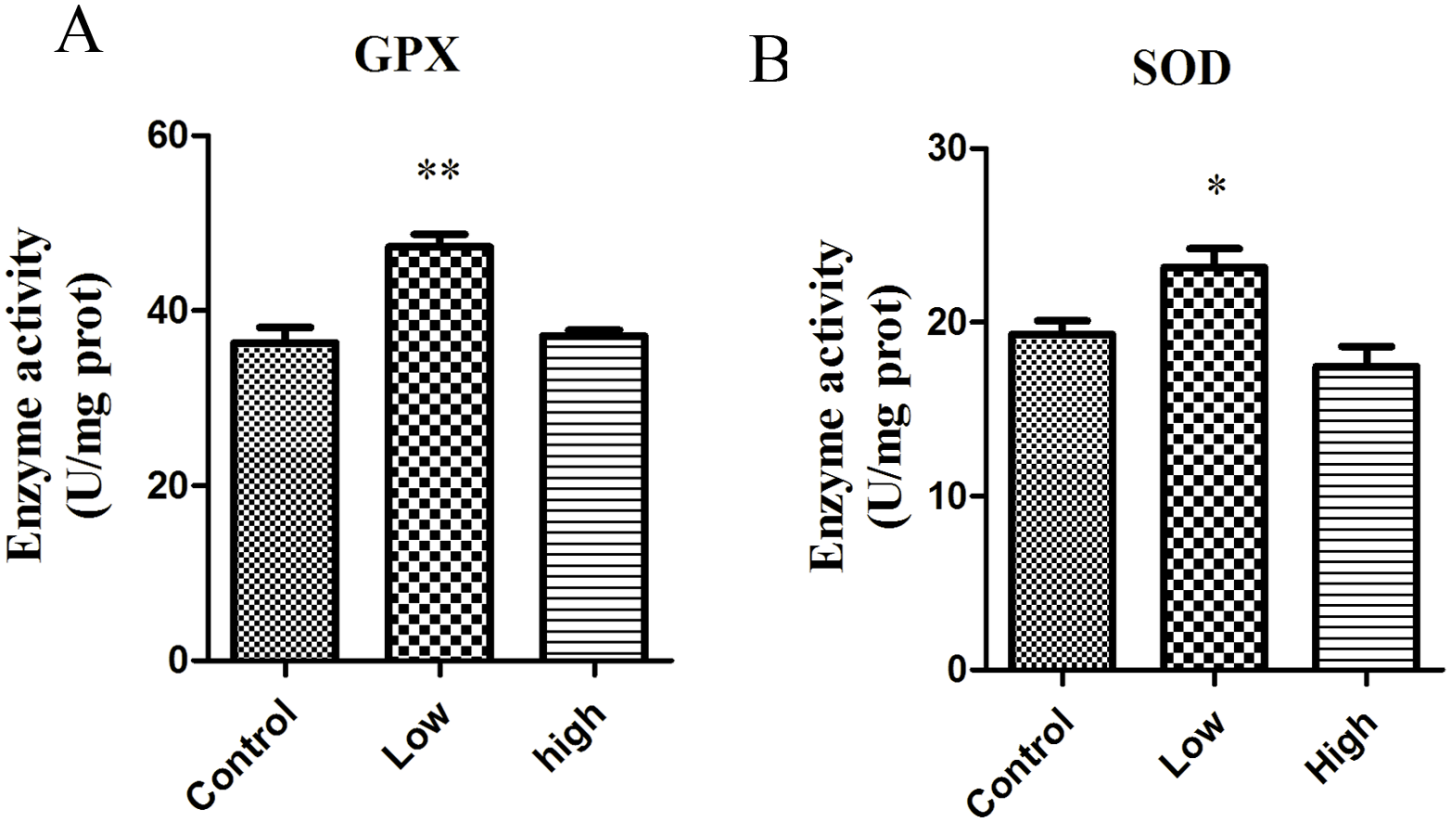
Effect of melamine on antioxidant enzyme activity in ovaries. In low group, GPX activity was significantly enhanced compare to control group. SOD activity was increased after exposure to 10mg/kg/d melamine compared to the control. Data show the means ± SE from at least three separate experiments. *P<0.05; ** P <0.01 versus control.

Since the expression of the key antioxidant enzymes was altered in melamine-treated oocytes, we further assessed the effects of melamine on the activity of GPX and SOD in mouse ovaries. As shown in Fig 3, the activity of GPX in the low-dose group was significantly higher than that of control group (P < 0.01), but the high-dose group showed no statistically significant difference compared to control group. For the activity of SOD, the low-dose group was remarkably higher than that of control group and there was no significant difference between high-dose group and control group.

### Effect of Melamine on the Ovary Morphology

**Fig 4:**
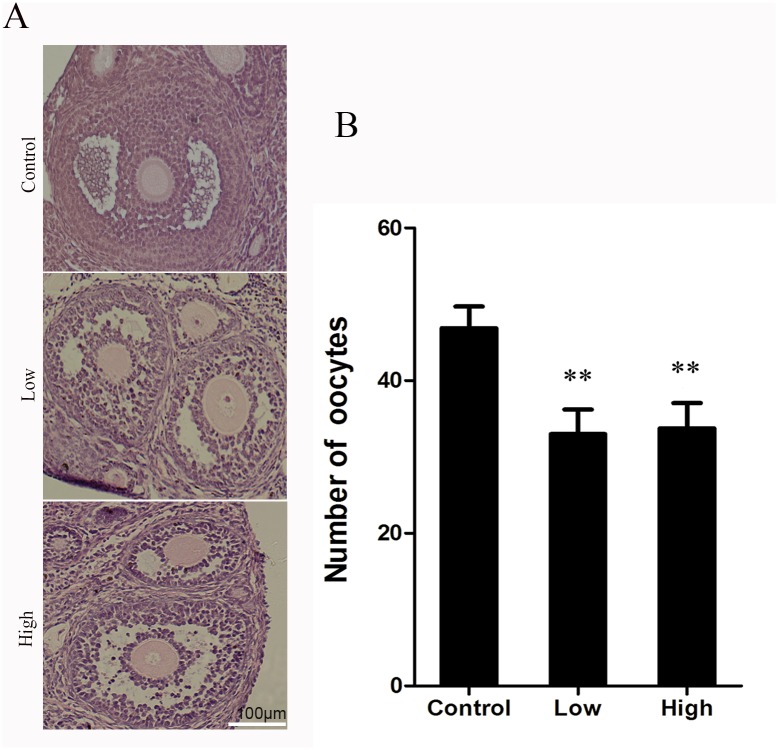
Effect of melamine on ovarian and histological analysis of the ovary. Hematoxylin and eosin staining was performed on paraffin sections of mouse ovary. The follicles showed normal cell associations with a lot of granulosa cells in the control group. The granulosa cells apoptosis and disrupted structure in the treatment groups. Data show the means ± SE from at least three separate experiments. **P <0.01 versus control.

To ask if there is any damage to the ovaries after melamine exposure, we performed the HE staining to observe their morphological changes and the number of oocytes. As presented in Fig 4, normal ovarian architecture and morphology with regular normal follicles were observed in the control group. In treatment groups, however, the atretic follicles increased in the ovaries, and the number of normal oocytes decreased compared to the control group.

## Discussion

Melamine is a nitrogen heterocyclic triazine compound that is widely used as an industrial chemical, and the toxicity of melamine was thought to be low in mammals. However, some studies have reported that animals and infants were affected by the food containing melamine [2,3,21]. Long-term exposure to melamine could lead to infertility and fetal toxicity in animals [22,23], and to induce oxidative stress to cause the oxidative disorders in the NRK-52e cells and PC12 cells [7,8]. Also, previous report by us has shown that melamine affects mouse oocyte maturation via cytoskeleton, apoptosis and autophagy induction, and epigenetic modifications [20].

In the present study, we further explored the possible mechanism regarding the effect of melamine on the follicle development and ovary formation. We found that melamine increases ROS levels, changes the expression and activities of SOD and GPX, and induces granulosa cell apoptosis as well as follicle atresia in the melamine-treated oocytes and ovaries.

Oxidative stress occurs when the production of oxidizing agents exceeds the antioxidant capacity of cellular antioxidant systems, which raises the physiological level of ROS, including free oxygen species and peroxides. Any disruption of this defense system will cause accumulation of ROS and lead to oxidative stress[24]. Oxidative stress plays an important role in endocrine disruptor-mediated dysfunction in the reproduction, and it is thought that ROS may serve as an early marker for toxicity evaluation [11]. ROS and antioxidants have been implicated in the regulation of follicle development, oocyte maturation, in both human and animal models [25,26]. In this study, the increased level of ROS after exposure to melamine indicated the excessive production of oxidizing agents in the oocytes.

GPX, SOD1 and CAT are the key players functioning in the process of oxidative stress. CAT transcripts cannot be detected in mouse GV oocyte, which is consistent with a previous report that CAT transcripts were not detected either in mice or in human oocytes, regardless of the stage of maturation [27]. Both SOD and GPX can be detected in oocytes and were altered by the treatment of melamine. SOD, GPX and non-enzymatic antioxidants play important roles in neutralizing ROS and protecting the oocyte and embryo from damage. SOD is responsible for dismutation of O2- to H_2_O_2_ and oxygen, and next, CAT and GPX reduce H_2_O_2_ to water and oxygen. In the present study, the significantly increased activity of GPX in response to 10mg/kg/d of melamine might be an early attempt to protect the ovaries from melamine-induced oxidative stress. However, the high dose of melamine group was significantly lower than the low dose group, which agrees with the report that the accumulated H_2_O_2_ can accelerate the degradation rate of GPX and results in the decrease of GPX activity [28]. In the lower concentration group GPX and SOD were activated. One possibility is that melamine-induced increases in both SOD and GPX activity would decrease accumulation of H_2_O_2_ and cause less damage in antral follicles than high group, but in high dose (50mg/kg/d) group, the antioxidant enzyme SOD which has been shown to play important roles in protecting the ovaries from oxidative damage began to upregulate the mRNA expression. However, the increased mRNA expression did not influence its activity, indicating that the increased mRNA expression would not immediately promote the protein expression or its activation. Therefore, with the reduced activity of SOD, the high dose of melamine overwhelmed the antioxidant system, and then led to a further increased ROS levels. The present data indicate that melamine induces oxidative stress mainly by suppressing the activity of SOD.

Some studies have shown that oxidative stress is one of the important factors for disruption of normal cell proliferation and apoptosis [29,30], and oxidative stress could induce granulosa cell apoptosis and further cause the follicular atresia, so that damage the oocyte quality [31,32]. The increased ROS level in melamine-treated oocytes prompted us to examine the follicle development and ovary morphology in melamine-treated mice. As expected, histochemical staining showed the apoptotic granulosa cells, decreased number of normal oocytes and atresic follicles in melamine-treated ovaries. These results revealed that melamine leads to granulosa cell apoptosis and follicular atresia through the oxidative stress pathway. Taken together, we demonstrated that melamine could induce the damage to mouse ovaries via the oxidative stress pathway involved in two regulators SOD and GPX. The detailed mechanism regarding how melamine disrupts the expression of SOD and GPX needs further investigation.

## Materials and Methods

### Ethic Statement

Animal care and use were conducted in accordance with the Animal Research Institute Committee guidelines of Nanjing Agricultural University, China. Mice were housed in a temperature-controlled room with proper darkness-light cycles, fed with a regular diet, and maintained under the care of the Laboratory Animal Unit, Nanjing Agricultural University, China. The mice were killed by cervical dislocation. This study was specifically approved by the Committee of Animal Research Institute, Nanjing Agricultural University, China.

### Animals and Treatment

The female 4-week-old ICR mice were kept at controlled conditions of temperature (20–23°C), illumination (12-h light-dark cycle) and had free access to food and water throughout the period of the study. Mice were randomly assigned to three groups (n = 40) and were each orally given 0, 10 or 50mg/kg/d of melamine dissolved in water for eight weeks.

The dosage for mice was chosen as previously study. About male mice, melamine had certain toxic effects on testes, especially when ingested in high concentration (50 mg/kg/day)[33]. In female mice, melamine had toxic effects on oocyte quality and fertility, especially in high concentration group (50 mg/kg/day)[20].

Oocytes were released from the ovaries by a brief exposure to M2 medium. The oocytes were then washed in M2 medium three times. Only oocytes with germinal vesicles were used for the experiment.

### Measurement of ROS and Confocal Microscopy

ROS labeled with GENMED Intracellular ROS Red Fluorescence Determination Kit (GENMED, Shanghai, China) for 30 min at 37°C in a 5% CO2 atmosphere and stained with Hoechst 33342 (10 μg/ml in PBS) for 10 min. Finally, oocytes were mounted on glass slides and viewed under a confocal laser scanning microscope (Carl Zeiss 700).

### Quantitative Real Time Polymerase Chain Reaction (qPCR)

Total RNA was extracted from oocytes using a Dynabeads mRNA DIRECT kit (Invitrogen Dynal AS), and first-strand cDNA was generated with a cDNA synthesis kit (Takara) using Oligo (dT) 12–18primers (Invitrogen). Primer sequences are shown in (Table 1).

**Table 1:** Sequences of primer sets used for gene expression analysis.

Gene name	Symbol	Forward primer	Reverse primer
Superoxide dismutase	Sod	5′- AAAGCGGTGTGCGTGCTGAA -3′	5′- CAGGTCTCCAACATGCCTCT -3′
Glutathione peroxidase	Gpx	5′- CCTCAAGTACGTCCGACCTG -3′	5′- CAATGTCGTTGCGGCACAC -3′
Catalase	Cat	5′- GCAGATACCTGTGAACTGTC -3′	5′- GTAGAATGTCCGCACCTGA -3′
Actin,beta	Actb	5′- GGGCACAGTGTGGGTGAC -3′	5′- CTGGCACCACACCTTCTAC -3′

### Assay of Antioxidant Enzyme Activity

Two mice per group were used to assay the activity of antioxidant enzymes. T-SOD activity was assayed by axanthine oxidase method based on the inhibition of nitrite formation from hydroxylammonium in the presence of O_2_—generators. Absorbance was measured at 550 nm. GPX activity was measured using DTNB method. Absorbance was measured at 412 nm. The decrease in absorbance was directly proportional to the GPX concentration. All detailed procedures were performed according to GSH-PX Determination kit and T-SOD Determination kit (Institute of Biological Engineering, Jiancheng, Nanjing).

### Histological Evaluation of Follicles

The ovaries were collected and fixed in 4% paraformaldehyde with 0.01 M phosphate-buffered saline (PBS) at 4°C overnight. After fixation, the tissues were dehydrated, embedded in paraffin, serially sectioned (5mm), mounted on glass slides, and stained with HE (haematoxylin and eosin). Ovarian sections were scanned under dot Slide-digital virtual microscope. Atretic follicles were identified using standard methods [34]; follicles were considered atretic if they contained more than 10 pyknotic nuclei, disorganized granulosa, a degenerating oocyte, or a fragmented oocyte nucleus.

### Statistical Analysis

The data were expressed as mean ± SE and analyzed by one-way ANOVA, followed by LSD’s post hoc test, which was provided by SPSS16.0 statistical software. The level of significance was accepted as p<0.05.

## References

[1] Ingelfinger JR (2008) Melamine and the global implications of food contamination. N Engl J Med 359: 2745–2748. 10.1056/NEJMp0808410 19109571

[2] Lam HS, Ng PC, Chu WC, Wong W, Chan DF, Ho SS et al (2008) Renal screening in children after exposure to low dose melamine in Hong Kong: cross sectional study. BMJ 337: a2991 10.1136/bmj.a2991 19097976 PMC2612581

[3] Parry J (2008) China's tainted infant formula sickens nearly 13,000 babies. BMJ 337: a1802 10.1136/bmj.a1802 18815175

[4] Cook HA, Klampfl CW, Buchberger W (2005) Analysis of melamine resins by capillary zone electrophoresis with electrospray ionization-mass spectrometric detection. Electrophoresis 26: 1576–1583. 15759307 10.1002/elps.200410058

[5] Xue M, Qin Y, Wang J, Qiu J, Wu X, Zheng Y et al (2011) Plasma pharmacokinetics of melamine and a blend of melamine and cyanuric acid in rainbow trout (Oncorhynchus mykiss). Regul Toxicol Pharmacol 61: 93–97. 10.1016/j.yrtph.2011.06.005 21723903

[6] Skinner CG, Thomas JD, Osterloh JD (2010) Melamine toxicity. J Med Toxicol 6: 50–55. 10.1007/s13181-010-0038-1 20195812 PMC3550444

[7] Guo C, He Z, Wen L, Zhu L, Lu Y, Deng S et al (2012) Cytoprotective effect of trolox against oxidative damage and apoptosis in the NRK-52e cells induced by melamine. Cell Biol Int 36: 183–188. 10.1042/CBI20110036 21939437

[8] Han YG, Liu SC, Zhang T, Yang Z (2011) Induction of apoptosis by melamine in differentiated PC12 cells. Cell Mol Neurobiol 31: 65–71. 10.1007/s10571-010-9554-4 20706782 PMC11498548

[9] Schafer FQ, Buettner GR (2001) Redox environment of the cell as viewed through the redox state of the glutathione disulfide/glutathione couple. Free Radic Biol Med 30: 1191–1212. 11368918 10.1016/s0891-5849(01)00480-4

[10] Rashid K, Sinha K, Sil PC (2013) An update on oxidative stress-mediated organ pathophysiology. Food Chem Toxicol 62: 584–600. 10.1016/j.fct.2013.09.026 24084033

[11] Valko M, Leibfritz D, Moncol J, Cronin MT, Mazur M, Telser J. (2007) Free radicals and antioxidants in normal physiological functions and human disease. Int J Biochem Cell Biol 39: 44–84. 16978905 10.1016/j.biocel.2006.07.001

[12] Cooke MS, Olinski R, Evans MD (2006) Does measurement of oxidative damage to DNA have clinical significance? Clin Chim Acta 365: 30–49. 16214123 10.1016/j.cca.2005.09.009

[13] Galazyn-Sidorczuk M, Brzoska MM, Jurczuk M, Moniuszko-Jakoniuk J (2009) Oxidative damage to proteins and DNA in rats exposed to cadmium and/or ethanol. Chem Biol Interact 180: 31–38. 10.1016/j.cbi.2009.01.014 19428343

[14] Mates JM, Segura JA, Alonso FJ, Marquez J (2012) Oxidative stress in apoptosis and cancer: an update. Arch Toxicol 86: 1649–1665. 10.1007/s00204-012-0906-3 22811024

[15] Agarwal A, Aponte-Mellado A, Premkumar BJ, Shaman A, Gupta S (2012) The effects of oxidative stress on female reproduction: a review. Reprod Biol Endocrinol 10: 49 10.1186/1477-7827-10-49 22748101 PMC3527168

[16] Hau AK, Kwan TH, Li PK (2009) Melamine toxicity and the kidney. J Am Soc Nephrol 20: 245–250. 10.1681/ASN.2008101065 19193777

[17] Neerman MF, Zhang W, Parrish AR, Simanek EE (2004) In vitro and in vivo evaluation of a melamine dendrimer as a vehicle for drug delivery. Int J Pharm 281: 129–132. 15288350 10.1016/j.ijpharm.2004.04.023

[18] Momose-Sato Y, Mochida H, Kinoshita M (2009) Origin of the earliest correlated neuronal activity in the chick embryo revealed by optical imaging with voltage-sensitive dyes. Eur J Neurosci 29: 1–13. 10.1111/j.1460-9568.2008.06568.x 19077122

[19] Wang W, Craig ZR, Basavarajappa MS, Hafner KS, Flaws JA (2012) Mono-(2-ethylhexyl) phthalate induces oxidative stress and inhibits growth of mouse ovarian antral follicles. Biol Reprod 87: 152 10.1095/biolreprod.112.102467 23077170 PMC4435432

[20] Duan X, Dai XX, Wang T, Liu HL, Sun SC (2015) Melamine negatively affects oocyte architecture, oocyte development and fertility in mice. Hum Reprod 30: 1643–1652. 10.1093/humrep/dev091 25924656

[21] Puschner B, Reimschuessel R (2011) Toxicosis caused by melamine and cyanuric acid in dogs and cats: uncovering the mystery and subsequent global implications. Clin Lab Med 31: 181–199. 10.1016/j.cll.2010.10.003 21295730

[22] Bock M, Jackson H (1957) The action of triethylenemelamine on the fertility of male rats. Br J Pharmacol Chemother 12: 1–7. 13413142 10.1111/j.1476-5381.1957.tb01352.x PMC1509654

[23] Generoso WM, Rutledge JC, Cain KT, Hughes LA, Downing DJ (1988) Mutagen-induced fetal anomalies and death following treatment of females within hours after mating. Mutat Res 199: 175–181. 3362157 10.1016/0027-5107(88)90243-6

[24] Agarwal A, Gupta S, Sekhon L, Shah R (2008) Redox considerations in female reproductive function and assisted reproduction: from molecular mechanisms to health implications. Antioxid Redox Signal 10: 1375–1403. 10.1089/ars.2007.1964 18402550

[25] Agarwal A, Gupta S, Sharma RK (2005) Role of oxidative stress in female reproduction. Reprod Biol Endocrinol 3: 28 16018814 10.1186/1477-7827-3-28 PMC1215514

[26] Al-Gubory KH, Fowler PA, Garrel C (2010) The roles of cellular reactive oxygen species, oxidative stress and antioxidants in pregnancy outcomes. Int J Biochem Cell Biol 42: 1634–1650. 10.1016/j.biocel.2010.06.001 20601089

[27] El Mouatassim S, Guerin P, Menezo Y (1999) Expression of genes encoding antioxidant enzymes in human and mouse oocytes during the final stages of maturation. Mol Hum Reprod 5: 720–725. 10421798 10.1093/molehr/5.8.720

[28] Lu X, Wang C, Liu B (2015) The role of Cu/Zn-SOD and Mn-SOD in the immune response to oxidative stress and pathogen challenge in the clam Meretrix meretrix. Fish Shellfish Immunol 42: 58–65. 10.1016/j.fsi.2014.10.027 25449371

[29] Chiu J, Dawes IW (2012) Redox control of cell proliferation. Trends Cell Biol 22: 592–601. 10.1016/j.tcb.2012.08.002 22951073

[30] Tilly JL, Tilly KI (1995) Inhibitors of oxidative stress mimic the ability of follicle-stimulating hormone to suppress apoptosis in cultured rat ovarian follicles. Endocrinology 136: 242–252. 7828537 10.1210/endo.136.1.7828537

[31] Corn CM, Hauser-Kronberger C, Moser M, Tews G, Ebner T (2005) Predictive value of cumulus cell apoptosis with regard to blastocyst development of corresponding gametes. Fertil Steril 84: 627–633. 16169395 10.1016/j.fertnstert.2005.03.061

[32] Jewgenow K, Heerdegen B, Muller K (1999) In vitro development of individually matured bovine oocytes in relation to follicular wall atresia. Theriogenology 51: 745–756. 10728999 10.1016/s0093-691x(99)00023-0

[33] Yin RH, Wang XZ, Bai WL, Wu CD, Yin RL, Li C et al (2013) The reproductive toxicity of melamine in the absence and presence of cyanuric acid in male mice. Res Vet Sci 94: 618–627. 10.1016/j.rvsc.2012.11.010 23261161

[34] Borgeest C, Symonds D, Mayer LP, Hoyer PB, Flaws JA (2002) Methoxychlor may cause ovarian follicular atresia and proliferation of the ovarian epithelium in the mouse. Toxicol Sci 68: 473–478. 12151644 10.1093/toxsci/68.2.473

